# A Culturally Grounded Method for Dialogue Between Indigenous Peoples and Researchers on Emerging Technologies: Lessons from the Gene Drive Context

**DOI:** 10.12688/gatesopenres.16383.1

**Published:** 2026-05-14

**Authors:** Neida Delia Andi Arimuya, Federica Bernardini, Pedro Jose Cabrera Andi, Gladman Chibememe, Florina Lopez Miro, Lucy Mulenkei, Aisatou Musa, Faith Nataya, Fabio Niespolo, Tony Nolan, Samantha O’Loughlin, Kamal Kumar Rai, Ndiaga Sall, Brian Tarimo, Jose Rafael Teran Maigua, María Yolanda Teran Maigua, Delphine Thizy, Carolina Torres

**Affiliations:** 1 Indigena Kichwa de la Amazonia Ecuatoriana, Amazonia, Ecuador; 2 Foro Internacional Indigena sobre Biodiversidad, FIIB, Quito, Ecuador; 3 Red de Mujeres Indigenas sobre Biodiversidad para America Latina y el Caribe, RMIB-LAC, Quito, Ecuador; 4 Imperial College London Department of Life Sciences, London, England, UK; 5 Indigena de la nacion Kichwa, Kichwa, Ecuador; 6 Chinhoyi University of Technology, Chinhoyi, Mashonaland West Province, Zimbabwe; 7 Indigena de la Nacion Kuna de Panama, Kuna, Panama; 8 Indigenous Information Network, Nairobi, Kenya; 9 Anura Ntabang woman CIG, Ntahbang, Cameroon; 10 Emerging Ag, Manitoba, Canada; 11 Liverpool School of Tropical Medicine, Liverpool, England, UK; 12 Nepal Indigenous Forum for Biodiversity, Kathmandu, Nepal; 13 ENDA Santé Sénégal, M'bour, Senegal; 14 Ifakara Health Institute, Ifakara, Morogoro Region, Tanzania; 15 Indigena Kichwa de la Sierra Norte, Sierra Norte, Ecuador; 16 The University of New Mexico, Albuquerque, New Mexico, USA; 17 Delphine Thizy Consulting, Brussels, Belgium; 18 Charles Darwin Foundation, Galapagos, Ecuador

**Keywords:** biotechnology, malaria, indigenous people, multi-stakeholder dialogue, stakeholder engagement, gene drive, genetic approaches, traditional knowledge, participatory method, synthetic biology, convention on biological diversity

## Abstract

This paper presents a participatory method for conducing a collaborative and culturally appropriate dialogue process between gene drive researchers and Indigenous Peoples and local communities. Coordinated by the Outreach Network for Gene Drive Research and representatives of the International Indigenous Forum on Biodiversity (IIFB), this dialogue aimed to build trust and facilitate mutual understanding and create a safe space for sharing traditional knowledge, rather than to reach decisions on the research or implementation of gene drive technology.

Over a three-year period, the dialogue evolved through multiple formats, recognising the specific needs to establish a meaningful and culturally appropriate dialogue between these two groups, while ensuring that Indigenous Peoples and local communities could share their traditional knowledge, traditions and innovations in a safe and trusted environment.

The method integrates key engagement principles – such as good faith, reciprocity, inclusivity, and respect for Indigenous Peoples and local communities’ knowledge systems – and describes how they were operationalised in practice. It provides a concrete example of applied engagement methodology in the context of gene drive and explores how these principles have influenced the dialogue’s format and the journey of both groups throughout this process, while also sharing some of the challenges they encountered.

This is not a theoretical review, but a joint account from practitioners from diverse backgrounds and interests, on how engagement methods can be implemented in real-world settings. The approach offers practical insights for designing sustained and scalable engagement strategies between scientists and Indigenous Peoples on complex and emerging science topics.

## Introduction

### The need for a culturally appropriate dialogue

In the research process, engaging rightsholders and stakeholders is paramount to ensuring the relevance, appropriateness, and ethical integrity of scientific and indigenous science processes. The practice has become increasingly integrated into common practice in public health ( Cunningham & Mercury, 2023)and environmental research ( Phillipson et al., 2012). This results from advocacy for better recognition and fair and genuine engagement of rightsholders and stakeholders to ensure their diverse perspectives and needs are considered. It adheres to ethical imperatives that ensure research is conducted with the culturally appropriate involvement and consent of those being studied or impacted. Additionally, it aspires to improve research quality and relevance by integrating rightsholders’ and stakeholders’ knowledge and expertise. For Indigenous Peoples and local communities, the request to be fully and effectively engaged and involved in research stems from the same: ensuring a recognition and consideration of their collective and self-determination rights – including their free, prior and informed consent – ensuring better quality and cultural appropriateness of the research, while ensuring respect and building trust. Still, the research process is often rooted in the history of cultural appropriation, marginalisation, exploitation and violations of human rights and of the rights of “Mother Earth and Mother Nature”. In the journey towards the recognition of their rights, Indigenous Peoples and local communities have fought for the respect of their knowledge systems, the consultation process, the free and prior informed consent for access to their knowledge and resources, and their full and effective participation throughout the research process ( Tsosie & Claw, 2019). The Convention on Biological Diversity (CBD) recognises Indigenous Peoples and local communities’ traditional knowledge, innovations and practices as science and the “promotion of their wider application with the approval and involvement of the holders of such knowledge, innovations and practices” in its Article 8j) ( Convention on Biological Diversity, 1992).

### The policy context for a dialogue on gene drive

In its programme of work on Article 8j adopted at COP15, the Conference of the Parties requests the Executive Secretary to “promote capacity-building and development for Indigenous Peoples and local communities at the national and local levels, on issues related to the Convention” ( Convention on Biological Diversity, 2022a). While many topics fall under this call, one has mainly been an area of attention considering its scientific development - synthetic biology, for which COP15 “call[ed] upon Parties and other stakeholders to facilitate broad international cooperation, technology transfer, knowledge-sharing […] for products of synthetic biology that are considered to be living modified organisms, and capacity-building on synthetic biology, taking into account the needs of Parties and Indigenous Peoples and local communities” ( Convention on Biological Diversity, 2022b). Modern biotechnologies more specifically, engineered gene drive technologies have been discussed both under the synthetic biology umbrella and as a living modified organism at the CBD and in international forums ( EFSA Panel on Genetically Modified Organisms et al., 2020; IUCN World Conservation Congress, 2016; IUCN World Conservation Congress 2020, 2021), and have been a topic of interest and concern for Indigenous Peoples and local communities representative organisations participating as observers in these processes ( International Indigenous Forum on Biodiversity (IIFB), 2022, 2024).

Gene drives are a naturally occurring phenomenon that biases the inheritance of a genetic trait, allowing its frequency to increase in subsequent generations ( Alphey et al., 2020; Burt & Crisanti, 2018; Burt Austin & Trivers Robert, 2007). Theoretically, this can be harnessed and reproduced through gene editing to rapidly transform a wild population to increase the proportion of individuals (‘frequency’) carrying the desirable traits. For example, it is envisaged to reduce the population of species that might endanger biodiversity (such as invasive alien species) ( Meiborg et al., 2023; Scudellari, 2019) or of insect species that transmit (‘vector’) agents that cause deadly diseases (e.g. malaria-transmitting mosquitoes) ( African Union, 2018; Burt et al., 2018; Eckhoff et al., 2017; Simoni et al., 2020). In the process of generating an organism carrying a gene drive, additional genetic material is added to the genome. Therefore, organisms containing gene drives are considered living modified organisms and, therefore, fall under the Cartagena Protocol ( Conference Of The Parties To The Convention On Biological Diversity Serving As The Meeting Of The Parties To The Cartagena Protocol On Biosafety, 2018), which approved new and voluntary additional guiding material for the risk assessment of these organisms at COP16. The CBD has also taken a decision clarifying the circumstances under which their release could be considered by Parties ( Convention on Biological Diversity, 2018).

Ongoing policy forums often provide researchers, Indigenous Peoples, and local communities with opportunities to meet and share their perspectives, particularly during negotiating sessions or side events. On these many occasions, it became clear to researchers working on gene drives and Indigenous Peoples and local communities representatives that a culturally appropriate dialogue was needed to ensure meaningful participation of Indigenous Peoples and local communities and for both groups to understand each other better within a framework of mutual respect and understanding. It was clear early on that the format of these policy fora – negotiations where observers intend to inform parties – was not necessarily the right place for this dialogue to take place and that specific activities needed to be co-developed with sufficient time to generate a quality and genuine dialogue based on trust, respect and inclusion.

### Purpose of this paper

This paper intends to present the dialogue method that was used by a group of gene drive researchers, coordinated through the Outreach Network for Gene Drive Research and a group of Indigenous Peoples and local communities experts and representative organisations, coordinated through the International Indigenous Forum on Biodiversity (IIFB). Academic papers have been published on some aspects of doing genetic engineering research on indigenous land and territories and on some specific aspects of rights-based decision-making in those contexts ( Barnhill-Dilling et al., 2020; George et al., 2019; Taitingfong, 2019). Similarly, indigenous researchers have published their perspective on gene editing in their context ( Hudson et al., 2019; Ratuva, 2009). This paper doesn’t aim to offer an alternative perspective on this topic or examine the respective positions on this research, including its potential benefits and risks. Rather, this paper aims to present how two stakeholder groups committed to advancing the objectives of the Convention and the Sustainable Development Goals have adapted an engagement method to build a dialogue on their challenges and opportunities. This is a practitioners’ paper aiming to provide a practical case study of how two groups from different parts of the world (Latin America and the Caribbean, Asia, Europe, Africa and Oceania) with different perspectives and priorities can come together to foster genuine and transparent dialogue and understanding.

## Method overview

The detailed inclusive and participatory methodology of this dialogue between researchers and right holders Indigenous Peoples and local communities will be described throughout the paper, highlighting the adaptations made to the process, in particular about oral, traditions, culture and social values, based on continuous exchanges between the two groups and, more particularly, thanks to the engagement of the focal points from each group.

The method can be summarised in five key phrases:

1. Initiation and partnership formation
2. Co-development of engagement principles
3. Adaptation to cultural and practical needs
4. Multi-format engagement activities
5. Iterative feedback and joint authorship

### Initiation and partnership formation

The engagement began with the introduction of gene drive researchers during an Indigenous Peoples and local communities caucus meeting at the Convention on Biological Diversity’s Subsidiary Body on Scientific, Technical and Technological Advice (SBSTTA) 22nd meeting in Montreal in 2018. Following that first meeting, a representative of the Indigenous Peoples and local communities expressed an interest in continuing the engagement for their caucus to be more informed on this topic. This process was part of a response to Indigenous Peoples and local communities demand to receive more information and capacity to be better equipped to engage in policy discussions on gene drive and, more broadly, on synthetic biology (though this process focused exclusively on gene drive). Indigenous Peoples and local communities have been participating in negotiations, in particular at the CBD, on synthetic biology and living-modified organisms, including those containing engineered gene drives, but have often expressed that they lacked adequate information to engage meaningfully. This dialogue did not intend to be the only source of capacity-building and development or information for Indigenous Peoples and local communities on this topic but to be one of the potential sources of information and to offer an opportunity for researchers to respond directly to Indigenous Peoples and local communities’ questions.

The gene drive researchers proposed to start a dialogue process, with no other objective than establishing a common understanding on this topic and a safe space for the groups to exchange. The agreement was originally to conduct a first series of online discussions with the use of an external and jointly approved facilitator. The original agreement did not plan for a long-term engagement or other activities, as both groups did not know whether this dialogue would succeed and what form it could take in the longer term.

### Co-development of engagement principles

Building trust, on co-developed engagement principles, was a foundational element to the success of the initial engagement between researchers and Indigenous Peoples and local communities. Prior to this dialogue process, the two groups mainly encountered each other in the CBD context, where discussions on synthetic biology and gene drive are very polarised, and therefore, the different participants might have come to the dialogue with some apprehensions and possible stereotypes or misconceptions. The researchers proposed initiating a formal dialogue, but concerns arose about how Indigenous Peoples and local communities in this polarised context might perceive this initiative.

Trust does require transparent communication and a commitment to mutual respect and collaboration. Still, in this context, creating a safe space for open and genuine dialogue appeared essential to building trust. An external facilitator, agreed upon by both groups, brought impartiality and objectivity to the process, mitigating power imbalances and allowing Indigenous Peoples and local communities participants to express themselves freely without fear of judgment. The Keystone Policy Center was hired by the researchers who had the financial resources for the project but with clear terms of reference that the process for the dialogue had to be driven equally by each group after careful engagement to elicit fundamental engagement principles, values and traditions that should be integrated into the dialogue set-up.

*“Generate greater trust between the parties. This requires transparent communication and mutual respect, a lot of commitment, and collaboration.”*

*Feedback and recommendation from a youth Indigenous Peoples representative from Latin America for future dialogue Indigenous Peoples and local communities*

**Figure 1.**
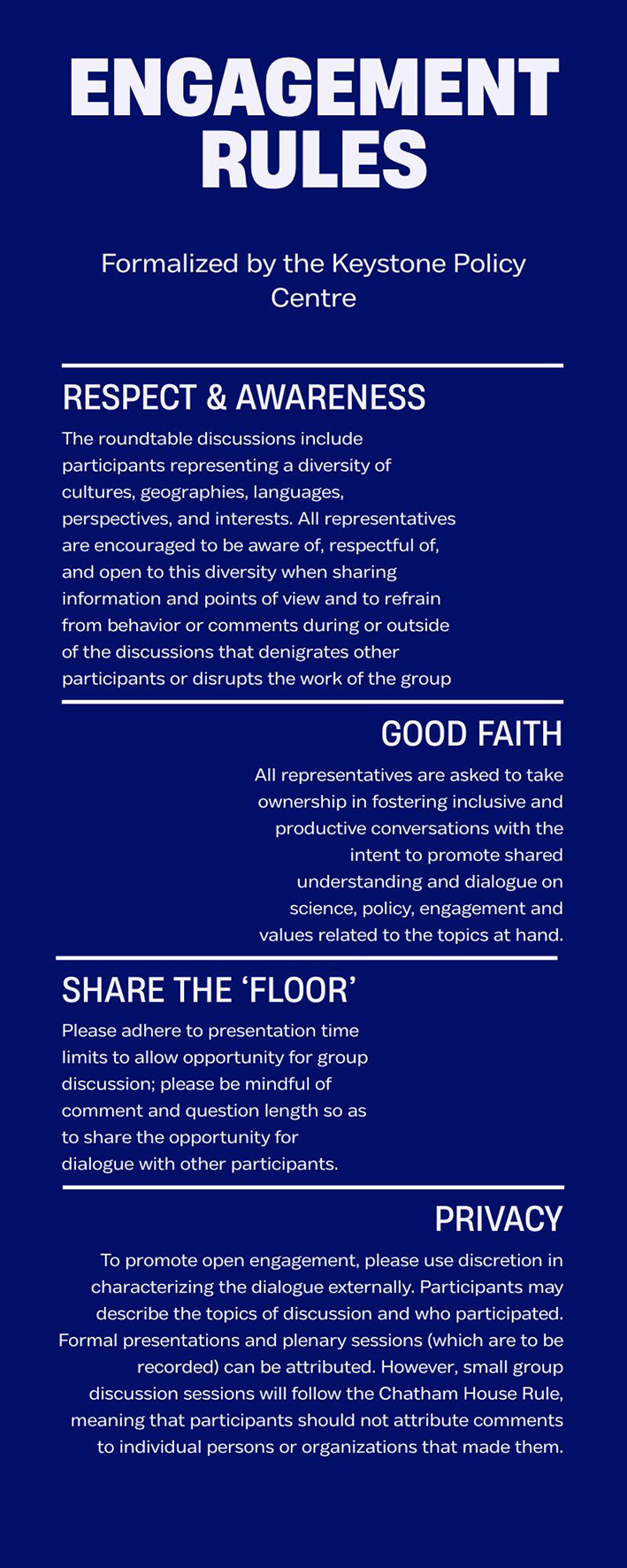
Rules of engagement agreed upon before the start of the formal dialogue – formalized by the Keystone Policy Centre.

As part of their facilitation efforts, the Keystone Policy Centre supported the co-development of basic rules of engagement ( Figure 1). These rules were formalised for the first interaction format (formal dialogue with webinars and consultations in 2021) and were critical to establishing the trust that guided the rest of the dialogue.

### Adaptation to cultural and practical needs

Building an effective and genuine dialogue between Indigenous Peoples and local communities and researchers required a nuanced understanding of cultural sensitivities and humility. The participants from each group came from different regions in the world – Latin America, Asia, Africa, Europe and the Pacific – and had diverse cultures, practices, and other knowledge and perspectives. Acknowledging participants’ diverse cultural backgrounds and value systems in this dialogue was essential for fostering respectful and meaningful relationships and engagement. Cultural sensitivity includes an openness to learning about and respecting the traditions and perspectives of Indigenous Peoples and local communities but also a willingness to adapt the engagement and dialogue processes accordingly. Researchers had to approach engagement with humility, acknowledging their knowledge deficit and refraining from imposing their prejudgements, assumptions, or agendas while building a dialogue model with the Indigenous Peoples and local communities.

*“The dialogue between researchers of gene drives and Indigenous Peoples and local communities opened a space of knowledge and communication within a framework of mutual respect, understanding, and acceptance. We started with an indigenous ceremony to ask for permission and guidance from our ancestors in this new activity. The Indigenous Peoples and local communities emphasised the respect for the individual and collective human rights of Indigenous Peoples, as well as due respect for the knowledge systems, cosmology, and epistemologies of the Indigenous Peoples and local communities.”*

From a youth participant of Indigenous Peoples and Local Communities from Latin America

(translated from Spanish)

### Multi-format engagement activities

From an original interaction in the form of online discussions facilitated by Keystone Policy Centre in 2021, the process evolved to a three-year dialogue with varied formats of interactions. The evolution of this process was mostly the result of regular coordination calls between 2021 and 2023 between representatives of the two groups, who were consulting other members of their group. The main feedback after the first interaction was a shared frustration that online dialogues were making trust-building and genuine interactions difficult, in particular for Indigenous Peoples and local communities who had more technical challenges with that format. When in-person meetings became possible again as the travel restrictions of the COVID-19 pandemic were being reduced, the groups started planning for a meeting on the sidelines of an existing policy meeting. The first in-person meeting took place on the sidelines of SBSTTA24 in Geneva and was followed in the same week by a lab visit at the Genomic Genetic and Biology Innovation Pole (Polo GGB) in Terni in 2022, taking the opportunity of the relative geographical proximity from the CBD meeting to this research facility.

*After the Geneva workshop, some key aspects were agreed upon for the continuation of the dialogue:*

*
“- Transparent transfer of information to the Indigenous Peoples and local communities to understand the risks and potential benefits of gene drives, with the greatest potential risks being the negative effects on biodiversity, culture, and traditional knowledge.*

*- Culturally appropriate and transparent communications to inform the Indigenous Peoples and local communities about the risks and benefits of gene drives, biodiversity issues, and ongoing and future projects.*

*- Effective participation of the Indigenous Peoples and local communities to form teams of indigenous and non-indigenous researchers and incorporate indigenous knowledge into the research on gene drives, respecting indigenous knowledge, know-how, and culture.*

*- Capacity building for the technological development of the Indigenous Peoples and local communities, emphasising the need for capacity building to improve understanding of genetic driver research and participation in research and consultation processes, highlighting that communication, education, and engagement are the cornerstone of capacity development.*

*- Exploring possibilities for scholarships and study grants for the Indigenous Peoples and local communities in universities so they can be trained in Synthetic Biology and related topics.*

*- Seeking slots for Indigenous Peoples in training to be held in Uganda or Latin America on gene drives.*

*- Continuing this type of dialogue so that the Indigenous Peoples and local communities receive first-hand information and clearly understand the aspects of Gene drives, their pros and cons concerning biodiversity.”*

From a man Indigenous Peoples representative from Latin America.

Building on the feedback from this first in-person interaction, Indigenous Peoples and local communities were invited to participate in the Pan-African Mosquito Control Association’s gene drive training sessions in 2022 and 2023, as a way to respond to their request for additional capacity building on the topic.

In 2022, both the gene drive researchers group and the Indigenous Peoples and local communities representatives felt the need to start a reflection on the joint collaboration. To foster a thoughtful evaluation of the initiatives undertaken and evaluate their outcomes, representatives of Indigenous Peoples and local communities and researchers organised a joint event at the Convention on Biological Diversity Conference of the Parties in Montreal in December 2022. This event served as a platform to publicly present the progress of ongoing dialogues and the various collaborative initiatives developed over the preceding two years. The outcomes of these initiatives were shared through detailed case studies, emphasising their successes in fostering open and transparent communication between the groups, as well as the challenges they faced. Both the perspectives of Indigenous Peoples and the Gene Drive Research community were thoughtfully presented, ensuring a balanced and inclusive discussion.

In 2023, an informal dialogue took place in Geneva during the Ad Hoc Open-Ended Working Group on Article 8(j), a moment to take stock of the dialogue and to discuss the possibility of a joint publication to share the experience of this dialogue.

Finally, this dialogue ended with the writing process for this paper, including a workshop held alongside the SBSTTA25 meeting in Nairobi in May 2024, which served to discuss these collaborative efforts.

These different formats contributed to a two-way capacity-building and knowledge-sharing process where information about the technology and its applications was shared with Indigenous Peoples but where researchers were also learning about Indigenous Peoples and local communities’ values, interests, concerns and rights. Throughout the process, Indigenous Peoples and local communities representatives took the opportunity to share information about their culture and traditions and their individual and collective rights. Particular attention was devoted to explaining the principles of their rights related to self-determination, consultation and free, prior and informed consent, including the right to veto. Recognising that the implementation and operationalisation of these rights vary between communities, the importance of consulting Indigenous Peoples and local communities about the approaches and protocols to establish the appropriate mechanisms was highlighted. Indigenous Peoples and local communities also shared their perspectives on Mother Earth and Mother Nature’s rights, and how this perspective impacts their relation to science and research.

*“This meeting was very important because they taught us about the technology they want to develop and consulted us about the ways of making decisions in Indigenous Peoples, as well as the rights of the peoples, in this case of Ecuador. We also shared concerns and fears due to the lack of information about gene drive development.”*

From a sister from Indigenous Peoples from Latin America

(translated from Spanish)

### Iterative feedback and joint authorship

When they decided to write a joint paper about this dialogue process, both groups agreed to focus the paper on dialogue method and what can be learned from this case study rather than on gene drive technologies, expectations and concerns. As pointed out by most Indigenous Peoples and local communities representatives the interactions – whether in the form of workshops, visits, trainings or informal exchanges – were too short to allow a thorough discussion about all potential benefits and risks of this technology, and often remained at a general level without entering in the specific characteristics of a specific gene drive organisms. Therefore, the intention was never to ask for any form of agreement, decision, or a specific list of concerns and expectations, but rather to engage in an exchange of information and perspectives.

The methodology used in this paper was inspired by the approach employed during the dialogue process between researchers and Indigenous Peoples and local communities. The objective was to co-develop a common outcome and a common ground, considering the perspectives and approaches of the two groups and their respective constraints. A core group of authors was established, gathering researchers and Indigenous Peoples and local communities involved in the dialogue process, who coordinated through calls and written exchanges for the overall definition of the paper’s objectives, structure, analysis and conclusions. An in-person workshop was also organised on the margin of the SBSTTA26 meeting in Nairobi in May 2024. Unfortunately, due to budget restrictions and competing agenda items, only two representatives from Indigenous Peoples and local communities could join this workshop, which was adapted to be a more informal discussion on the dialogue process and the initial draft paper.

A broader group of authors was included in the paper’s preparation and review, providing inputs and sharing their traditional knowledge. This included the set-up of a *WhatsApp* group, considered an easier channel for exchange, where the latest developments were shared in Spanish, English and French. The final manuscript was shared with all co-authors for input. Co-authors from both groups provided comments on the text. Others less used to this process of academic writing shared their inputs in a separate written form in Spanish, which was then directly integrated into the manuscript text or in the form of quotes to present those perspectives in their original form. Final discussions were held in person during the Conference of the Parties of the Convention on Biological Diversity that took place in October 2024 in Cali, Colombia, where several co-authors were present.

This tiered approach takes into account some of the constraints of peer-review publications – English language proficiency and adherence to academic writing conventions – but allows, at the same time, the integration of traditional knowledge and perspective in a meaningful way. The text was translated into Spanish to support the participation of some of the co-authors, and a text box was also used to provide an opportunity to share traditional knowledge perspectives without having to constrain it to the format imposed in the main text of a peer-reviewed paper.

The authors acknowledge the systemic limitations of the peer-reviewed publication process in accommodating the diverse perspectives and contributions of Indigenous Peoples and local communities. Beyond logistical challenges such as limited access to research funding, language barriers, and adherence to standardised writing formats, deeper ethical and cultural considerations exacerbate the hurdles Indigenous Peoples and local communities face in publishing in peer-reviewed journals. Notably, the conventional authorship paradigm, which attributes knowledge creation to individual authors, diverges from Indigenous and traditional knowledge systems, where knowledge is collectively generated, owned, and safeguarded, and orally transmitted from one generation to the next through customary systems and cultural processes. In response to this discrepancy, the authors addressed this issue by advocating for including the nations, people or community to which they belong in the affiliation, thus acknowledging their integral role in knowledge production and dissemination.

A keen awareness of power dynamics and ethical considerations was paramount in this paper’s writing process and the broader dialogue initiative. The researchers had funding resources designated for the paper’s development, placing them in relative superiority vis-à-vis the Indigenous Peoples and local communities. Consequently, attention was devoted to establishing mechanisms for the consultation and validation of each stage of the paper’s development and finalisation, with a deliberate focus on ensuring that Indigenous Peoples and local communities, voices could be heard, acknowledged, and integrated. To facilitate Indigenous Peoples and local communities’ meaningful participation, funding allocations from the project budget were earmarked to support the attendance of Indigenous Peoples and local communities’ representatives at writing workshops. However, in acknowledgement of the imperative to uphold the autonomy and agency of Indigenous Peoples and local communities representatives, no financial compensation was offered for their time or contributions throughout the three-year process, whether about the paper itself or the broader dialogue initiative. This approach was adopted to safeguard the integrity and authenticity of Indigenous Peoples and local communities’ perspectives and decisions, fostering a collaborative, fair and equitable partnership between researchers and Indigenous Peoples and local communities representatives.

## Tools and practical considerations

Ensuring the accessibility of this dialogue was paramount to its success. Accessibility hinged on a variety of tools and processes, including interpretation and translation services; however, practical accessibility issues also posed significant challenges. For Indigenous Peoples and local communities and researchers from Low- and Middle-Income Countries, accessing the online dialogue platform and maintaining a stable internet connection proved arduous and unequal. Despite efforts to enhance accessibility through interpretation services and accommodating various time zones, these practical challenges significantly hindered stakeholder participation.

Similarly, attending physical meetings presented obstacles, with the visa application process often proving cumbersome. For Indigenous Peoples and local communities members, travel within countries was required, while for some West African researchers, travel between countries was necessary, resulting in complex logistical coordination and additional costs. These accessibility considerations are crucial to bear in mind when planning such dialogues, as they represent substantial financial and human resource burdens that necessitate thorough planning and preparation.

Efforts to increase accessibility and dialogue required continuous coordination between the two groups during the three years of the process. This was achieved through exchanges between a small group, including an Indigenous woman from Latin America, who coordinated the broader dialogue effort to maintain open communication channels. This coordination was facilitated through regular calls and information exchanges, which were crucial for maintaining interest in the dialogue, outlining its next phases, and gathering transparent and constructive feedback to inform subsequent phases.

Respecting the diverse culture, customary laws, biocultural procedure and traditions of Indigenous Peoples and local communities, and the diversity of stakeholders participating in these dialogues requires adapting the language for the various exchanges. The first step was to ensure that all written materials – such as those prepared before the first online dialogues – and all verbal interactions were interpreted. The languages chosen were English and Spanish, considering the countries of origin of the Indigenous Peoples and local communities’ representatives. However, translation and interpretation are only part of the language adaptation needed for such a discussion. Researchers had to ensure that they adapted their language to the knowledge and interests of Indigenous Peoples and local communities to engage meaningfully on this topic. For instance, during the consultation meetings facilitated by Keystone Policy Center in 2021, the materials provided by the researchers on the science of gene drive were simplified. However, they were judged still too complex and challenging to engage with. In subsequent meetings, such as the dialogue organised in 2022 in Geneva and Terni, the science of gene drive was explained differently through more illustrative and oral stories, and the laboratory visit to Terni was a direct response to the need to see and experience the scientific context directly, instead of apprehending it through theoretical concepts. The quality of dialogue improved significantly(which was demonstrated by an increased participation of Indigenous Peoples and local communities through interventions to ask questions, express their perspectives about this technology) and Indigenous Peoples and local communities representatives who had participated in various interactions expressed that these approaches supported their understanding of gene drive mechanisms.

*“The scientists in charge of the presentations were very patient and spoke to us in quite comprehensible language.”*

From a sister from Indigenous Peoples from Latin America (translated from Spanish)

## Results and observations

### Success factors

This dialogue was guided by common engagement principles that helped foster respect, trust, solidarity and collaboration. These principles were the backbone of this dialogue, ensuring that the initiative maintained some consistency throughout the years and activities.

Good faith and collective ownership were fundamental principles underpinning this initiative. Both groups came to this dialogue in good faith, demonstrating a genuine commitment to collaborate, listen and adapt without expectations to convince other participants to take a particular position or change their perspective. To anchor this principle, it was made clear from the beginning and at various steps of the process that this dialogue did not aim to seek or obtain any form of consent for the research or to make any form of joint statement concerning the ongoing policy forums. Collective ownership was ensured through the design of collective activity and decision-making processes for each activity. Although there was an inequality of power in the funding aspect, the researchers’ resources enabled the implementation of mechanisms that ensured Indigenous Peoples and local communities were respected and considered equal partners, fostering a sense of ownership and investment in the outcomes.

Reciprocity and mutual benefit constituted indispensable principles in fostering a balanced and constructive dialogue, highlighting the significance of equitable exchange and collaboration. Both parties sought to ensure that the dialogue yielded tangible benefits, including augmented capacity-building opportunities, access to firsthand information, heightened decision-making autonomy for Indigenous Peoples and local communities and researchers, facilitation of meaningful engagement with a pivotal rights-holders and stakeholder group, enhanced information-sharing practices and meaningful engagement approaches. Reciprocity was the bedrock for establishing trust and fostering a sense of partnership, enabling each partner to contribute their expertise and knowledge while feeling assured of recognition and respect by the other party.

*“For me, it was a rewarding experience to feel that there is an interest in uniting or consulting both sciences: scientific and traditional.”*

From a sister participant of Indigenous Peoples from Latin America

(translated from Spanish)

Respect for Indigenous and traditional knowledge and traditions was paramount for the success of this dialogue. Researchers had to approach engagement with Indigenous Peoples and local communities with humility, good faith, and actively taking the consent of Indigenous Peoples and local communities on how to incorporate their traditional knowledge-seeking practices and innovations into the dialogue, recognising its relevance and applicability to this process. A symbolic example of this integration was initiating each meeting with a traditional ceremony led by an indigenous representative from Latin America. The respect for Indigenous knowledge and traditions was not limited to the opening ceremony but also the allocation of dedicated sessions on the agenda for Indigenous Peoples and local communities to share their knowledge and practices. For example, at the Geneva workshop in 2022, four Indigenous Peoples and local communities representatives (three from different geographical areas of Ecuador and one from Nepal) shared their knowledge and tradition, ranging from an overview of the traditional culture of the Indigenous youth perspective on gene drive to the overall priorities of Indigenous Peoples and local communities at the CBD, and the Indigenous Peoples and local communities’ overall perspective on gene drive.

The final principle was to find a balance between inclusiveness and representativeness. This dialogue process was designed as an iterative process to be inclusive of Indigenous Peoples and local communities interested in this process, who come from different perspectives, while ensuring some representativity of the contexts where gene drive is currently being envisaged for its first applications. The tension between inclusiveness and representation is significant when discussing gene drive in a forum like the CBD, as many gene drive projects focus on applications for Africa. In contrast, Indigenous Peoples and local communities’ representation at the CBD includes a much broader set of Indigenous Peoples and local communities, with, for instance, a large contingent from Latin America. The dialogue process strives for inclusiveness but has to question this inclusiveness for representation and relevance for specific activities and areas. For instance, for the participation in the Pan-African Mosquito Control Association training on Gene Drive, due to the focus on applications for malaria and for Africa and limited funding availability, the activity was restricted to African Indigenous Peoples and local communities.

### Challenges

Facilitating effective knowledge exchange emerged as a notable challenge during this initiative. Through experiential learning, it became evident that storytelling and visual and hands-on experiences, such as laboratory visits, constituted the most efficacious means of engaging stakeholders on gene drive science. Notably, an opportunity for such engagement was seized alongside an existing meeting — SBSTTA24 in Geneva — thereby ensuring manageable logistics and costs. However, it is imperative to acknowledge the inherent difficulty in replicating and scaling this approach to accommodate larger groups, particularly given that policy meetings are not consistently held near gene drive research laboratories.

Moreover, the quality of dialogue is intricately linked to the trust and relationships cultivated among participants over time and the cumulative knowledge accrued throughout the engagement process. Consequently, there arises a pressing need to maintain a degree of stability among dialogue participants to facilitate the generation of meaningful outcomes. By prioritising continuity and fostering trust among participants, stakeholders can collectively contribute to advancing informed decision-making and policy development in gene drive research.

Recognising diversity within the group of researchers and Indigenous Peoples and local communities was critical for understanding the context and building trust. The needs vary between the groups and within the groups as the Indigenous Peoples and local communities are not homogenous entities, just as researchers are not; they are composed of individuals with differing priorities, aspirations and experiences. For instance, in this dialogue, some participants working in Africa – researchers and the Indigenous Peoples and local communities – were more focused on applications of gene drive for malaria (as Africa bears 90% of the burden of this disease) ( World Health Organization, 2023), while for other participants, such as the Indigenous Peoples and local communities from Latin America, this research and its implications might be less directly relevant in their context, due to a lower prevalence of malaria in LATAM. Their interest might be more focused on gene drive applications for the fight against dengue or other vector-borne disease, invasive alien species or agricultural applications. Acknowledging and allowing for different priorities within the dialogue entails adapting the format to maintain a cohesive process while ensuring flexibility and openness to embrace these differences.

The Indigenous Peoples and local communities expressed the need for capacity building and development for different actors and diverse levels in synthetic biology, including gene drive technologies, which presents a multifaceted challenge that cannot be adequately addressed through a singular approach. This limitation arises from the inherent constraints in the scope of technologies and perspectives covered by existing initiatives and the inherently restricted number of participants engaged in such dialogues.

Nonetheless, throughout this comprehensive process, the coordination group diligently endeavoured to amplify local voices and augment the impact of this dialogue. This approach was predicated on the belief that the dialogue would be significantly enriched by including Indigenous Peoples and local communities representatives who might not participate as actively in global policy deliberations. Moreover, it was anticipated that Indigenous Peoples and local communities’ representatives would serve as conduits of knowledge within their communities, thereby fostering broader engagement and consultation on gene drive technologies at the grassroots level. Through this concerted effort to amplify Indigenous Peoples and local communities’ voices and ensure their full and effective participation, the coordination group sought to foster a more inclusive and impactful dialogue that reflects the diversity of perspectives and experiences inherent to Indigenous Peoples and local communities’ settings and environs, livelihoods, lands, territories, waters and resources.

The Indigenous Peoples and local communities requested to have access to opportunities to exchange knowledge and develop their capacity to understand gene drive, which was expressed particularly strongly during the in-person meetings in Geneva in 2022. In response, a partnership was established with the Pan-African Mosquito Control Association (PAMCA). PAMCA organises an annual conference on mosquito control in Africa and alongside this conference since 2017 has convened a three day short course on gene drive for African biologists and mosquito control professionals. This course was designed for participants with a strong scientific background, and considering the feedback provided after the first instances of knowledge exchange, the decision was made to organise a Foundation Course to introduce Indigenous Peoples and local communities representatives to the subject before they would join the rest of the course. The Foundation Course included modules on basic genetics (introducing concepts such as DNA, genes, and heredity), conventional mosquito control tools, introduction to the concepts behind gene drive, and how a gene drive mosquito is made. The course was designed to assume no previous knowledge of genetics or science generally, where possible employing pictures, videos and physical media, and including time for questions and discussion after each talk. After feedback from the first course in 2022, a module was added on regulatory and stakeholder engagement considerations. Two African Indigenous People and Local Communities participated in this training in 2022 and were invited to attend the rest of the PAMCA conference if interested. In 2023, a further two African Indigenous People and Local Communities’ representatives participated in the Foundation Course, which was also expanded to include other non-scientists such as African authorities representatives. Two of the participants were from Francophone countries, and interpretation was provided. Due to resource contraints but also feedback from the 2022 participants about the fact that this main course was too technical, in 2023, occasion the Foundation course was operated as ‘stand alone’ and not as an adjunct to the main gene drive course. After each course a questionnaire was provided to all participants to enable them to give anonymous comprehensive feedback on each part of the course. The feedback received was overall positive about the initiative, with participants mentioning “a truly positive experience” or “valuable insights and knowledge throughout the program” in their feedback. However, a common criticism was shared by participants that even though trainers were “highly knowledgeable, engaging, and patient, making complex topics easier to grasp”, the density of the content and the fact that the training was too short to cover those topics, limiting, therefore, the time to “digest” and leaving “questions unanswered”. The feedback and subsequent discussions revealed a strong interest in similar activities, but the format still requires further thinking to make the content and process accessible to Indigenous Peoples and local communities considering their cultural times, working rhythms and Indigenous epistemologies and languages.

*“However, it might also be advisable for the training sessions to be longer and to be carried out with a lot of respect, since in the worldview of Indigenous peoples we are all part of the same universe.”*

From a sister representative of Indigenous Peoples from Latin America.

It is, however, interesting to note that similar feedback was provided by the participants to the overall gene drive training organised by PAMCA (to which the Indigenous Peoples and local communities also attended in 2022), where several comments ask for “More time allocations for discussions”. This demonstrates that the issue of the format and density of content is not unique to Indigenous Peoples and local communities participants but rather to the whole training, considering the complexity of gene drive as a subject and the attempt to cover the biological, regulatory and engagement aspects in one training.

## Discussion

**Figure 2.**
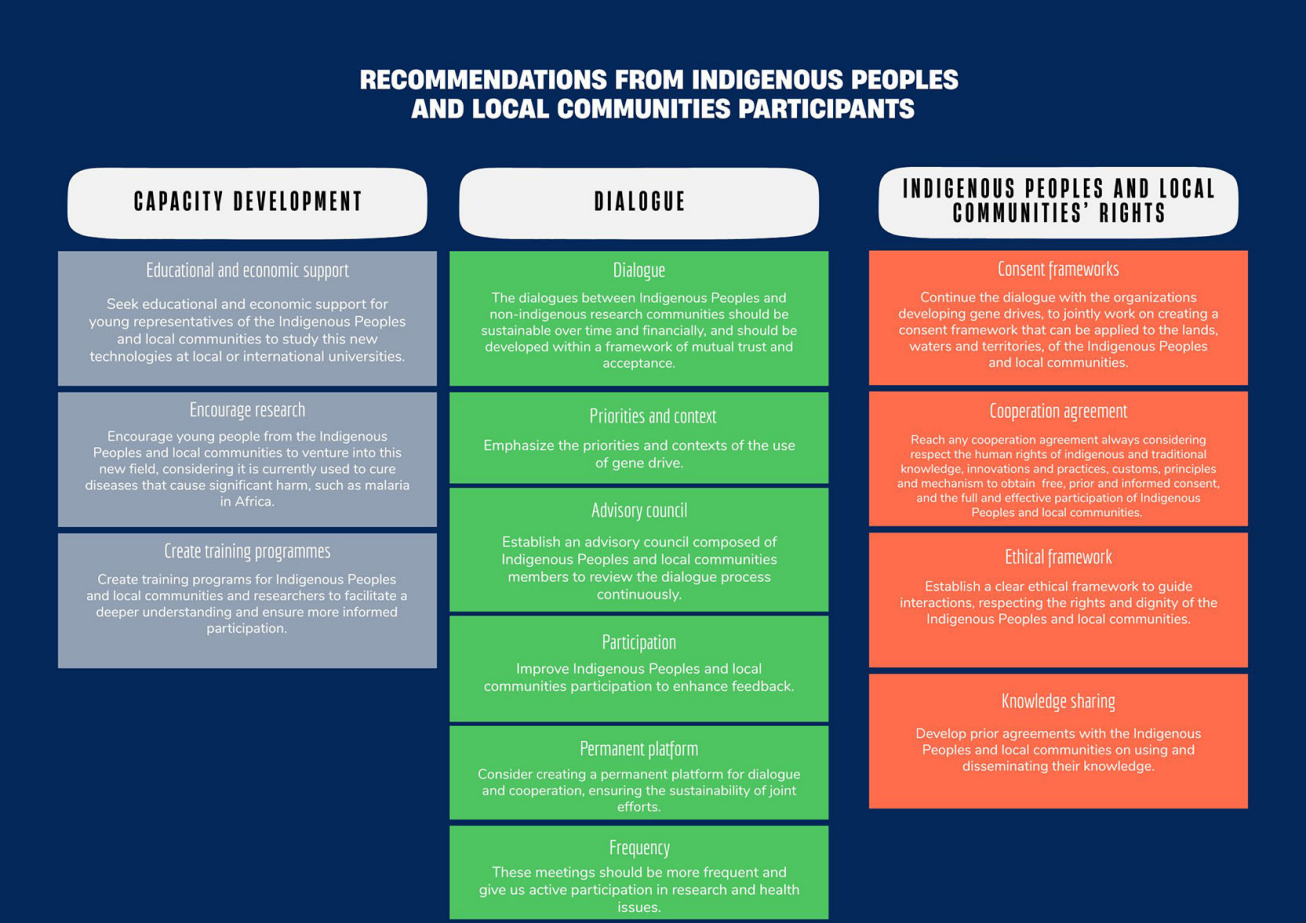
Recommendations from Indigenous Peoples and local communities participants to the dialogue.

The preparation of this paper allowed for a deeper stock-taking exercise, assessing in a more structured and organised way what had been achieved, the challenges, and the expectations of both groups. The discussions to prepare this paper highlighted how central capacity building and development were and, at the same time, how challenging they were. This is reflected by the importance of capacity building in the recommendations for future processes ( Figure 2). The first challenge arises from the complexity of the topic and the limited time available. As experienced by some gene drive research projects that have been engaging Indigenous Peoples and local communities about the topic for years, meaningful engagement and capacity development require time and sustainable engagement through several iterations ( Pare Toe et al., 2021, 2022). This is possible in a research setting where community members and researchers are working in the same context and where the potential impact of the research creates a motivation for community members to engage in this dialogue process over a long period and through numerous iterations ( Barry et al., 2020).

Engaging Indigenous Peoples and local communities representatives from ancestral traditional territories where the research is not taking place is challenging because the topic interests these representatives, while not directly or immediately impacting their rights or activities. Therefore, the intensity of engagement cannot be as high, and reaching a similar level of knowledge exchange is more complicated.

The second challenge concerns the scalability and sustainability of this effort. A small group of researchers and Indigenous Peoples and local communities representatives initiated the dialogue process and kept it active throughout the period. Considering the limited resources available for this dialogue, it had to focus on a small number of participants from both groups, who cannot be viewed as representatives of the broader group despite the efforts to strive for representativeness and inclusivity. A participant expressed the challenge of sharing the learning back in her community, considering the complexity and the fact that gene drive might not yet be an area of interest for that community. She mentioned sharing her experience, showing her pictures, and hearing community members raise similar questions. The objective of these capacity-building activities was not for the participants to become trainers in their community. Still, some wanted to share more but did not feel sufficiently equipped. The question of sustaining the interest of those engaged in this dialogue was also raised ( Figure 2). At the Nairobi informal discussion on the paper, the idea to create a more institutional network for the training alums emerges, as well as to have a forum to continue exchanging with the researchers, which would allow Indigenous Peoples and local communities to share their perspective, knowledge but also to have access to academic knowledge and responses to their questions.

These challenges are, to some extent, related to the framework and scope of this dialogue. The dialogue emerged as a response to a need for more dialogue between researchers and Indigenous Peoples and local communities observed in global forums such as the Convention on Biological Diversity and the International Union for the Conservation of Nature. In both cases, the forum brings together rightsholders and stakeholders from around the world who, although not intervening in the same territories, share an interest in a similar topic, despite differing perspectives, concerns, or hopes. The quality of the dialogue and knowledge exchange is affected by these circumstances because, as highlighted before, participants might not be intervening in similar territories or could have a marginal interest in this topic and, therefore, not necessarily have the time to commit to a sustained dialogue effort, considering other essential issues for them.

Acknowledging this situation and the fact that engagement with Indigenous Peoples and local communities might be more structured and meaningful at local levels where the research is taking place, the idea emerged of organising meetings – physically or virtually – between Indigenous Peoples and local communities representatives who participated in this dialogue process and who are active in international forum Indigenous Peoples and local communities where gene drive research is currently taking place. This proposal aims to further knowledge exchange on gene drive technologies by involving Indigenous Peoples and local communities that might have participated in this research for years, and learning more about the dialogue process there. It would also offer these communities an opportunity to hear the perspectives of Indigenous Peoples and local communities’ representatives who actively advocate for their rights related to these technologies at the global level.

## Conclusion

As evidenced by the dialogue process explored in this paper, effective engagement with the Indigenous Peoples and local communities requires a nuanced understanding of cultural sensitivities, humility, and a commitment to building trust and relationships based on mutual respect. Practical considerations, such as ensuring accessibility to dialogue platforms and addressing logistical challenges, are equally critical in fostering meaningful, fair and genuine engagement. Moreover, the dialogue process highlighted the importance of adapting communication approaches to accommodate diverse perspectives and prioritise inclusivity.

Central to the success of the dialogue initiative was the adherence to common engagement principles, including good faith, transparency, collective ownership, reciprocity, and respect for Indigenous and traditional knowledge, innovation, practices and cultural traditions. By upholding these principles, researchers and Indigenous Peoples and local communities stakeholders established a collaborative and equitable dialogue framework that facilitated knowledge-sharing, capacity building and development.

Moving forward, similar dialogues between researchers and Indigenous Peoples and local communities must be encouraged and supported, whether on gene drive or other emerging technology topics. Parties to the CBD should consider investing in similar initiatives as part of ongoing efforts to address power imbalances, promote inclusivity, and ensure that Indigenous Peoples and local communities’ perspectives are integrated into research processes and decision-making forums. By doing so, stakeholders can collectively contribute to advancing sustainable development goals and promoting social and environmental justice.

## Ethics statement

This paper does not report on research involving human participants, their data, or biological samples. Instead, it describes a dialogue process between two groups, focusing on the methodology and outcomes of that process. No personal or health-related information was collected, stored, or analyzed, and no interventions were undertaken with individuals for research purposes.

As such, the work does not meet the definition of research involving human subjects under the Declaration of Helsinki and corresponding national/international guidelines. Therefore, institutional ethics committee approval and informed consent were not required.

## AI use

This paper was written without the use of Generative AI, however the grammar and syntax were checked with Grammarly 1.131.1.0 version.

## Data Availability

No datasets were generated or analysed for this manuscript. The work is based on a description of a process rather than data collection. As a result, no dataset is available.
